# Bio-Inspired Polymeric Solid Lipid Nanoparticles for siRNA Delivery: Cytotoxicity and Cellular Uptake In Vitro

**DOI:** 10.3390/polym16233265

**Published:** 2024-11-24

**Authors:** Keelan Jagaran, Saffiya Habib, Moganavelli Singh

**Affiliations:** Nano-Gene and Drug Delivery Laboratory, Discipline of Biochemistry, University of KwaZulu-Natal, Private Bag X54001, Durban 4000, South Africa; 215055447@stu.ukzn.ac.za (K.J.); saffiya.habib@gmail.com (S.H.)

**Keywords:** neurological disorders, nanomedicine, solid lipid nanoparticles, gene delivery, siRNA, *Ginkgo biloba*, biological

## Abstract

Nanomedicine has introduced strategies that provide precise diagnosis and treatment with fewer side effects than traditional therapies. Treatments for neurodegenerative disorders, including Parkinson’s disease, are palliative, necessitating an innovative delivery system with a curative function. This study investigated a solid lipid nanoparticle (SLNP) system’s ability to bind and safely deliver siRNA in vitro. SLNPS were formulated using sphingomyelin and cholesterol, with *Ginkgo biloba* leaf extract (GBE) incorporated to enhance biocompatibility and neuroprotection. Poly-L-lysine (PLL) functionalization ensured successful siRNA binding, safe transport, and protection from nuclease degradation. SLNPs were physicochemically characterized, with binding and protection of siRNA assessed using agarose gels. Cytotoxicity, apoptotic induction, and cellular uptake studies were undertaken in the human neuroblastoma (SH-SY5Y) and embryonic kidney (HEK293) cells. The GBE-PLL-SLNPs had an average size of 93.2 nm and demonstrated enhanced binding and protection of the siRNA from enzyme digestion, with minimal cytotoxicity in HEK293 (<10%) and SH-SY5Y cells (<15%). Caspase 3/7 activity was significantly reduced in both cells, while efficient cellular uptake was noted. The present study provided a solid basis as a proof of principle study for future applications of the potential therapeutic in vitro, promising to address the unmet medical needs of patients with neurological disorders.

## 1. Introduction

The evolution of nanomedicine has led to innovative methods for diagnosis and therapy while minimizing the adverse effects caused by traditional medicine. Lipid nanocarriers have considerable advantages due to their biodegradability, scale-up capacity, low toxicity, biocompatibility, and the potential to deliver lipophilic and hydrophilic drugs in a targeted and controlled manner [1,2]. Solid lipid nanoparticles (SLNPs) are composed of a solid physiological lipid (at room or body temperature), a surfactant, and water [3]. SLNPs have been reported to be biodegradable and biocompatible, with the ability to protect the bound therapeutic. These favorable characteristics rival liposomes, which are among the most popular lipid-based systems used in gene delivery [4,5]. SLNPs usually range from 10 to 1000 nm in diameter and possess pivotal characteristics relating to their ease of cellular infiltration [6].

SLNPs have proved to possess more advantages over polymeric NPs. They are more cost-effective to synthesize and can be scaled up for various applications [7]. Furthermore, their synthesis avoids potentially harmful organic solvents, rendering them less toxic for medical use [8]. They have high evasive properties and can prevent reticuloendothelial system (RES) uptake, bypassing the liver and spleen and preventing filtration [9]. SLNPs have a high encapsulation rate, encapsulating lipophilic and hydrophilic molecules while maintaining their stability [10]. Similar to other NPs, SLNPs, when conjugated to an appropriate ligand, can improve the targeted and sustained release of drugs and genes [4].

Various phospholipids may be employed in the synthesis of SLNPs. Sphingomyelin is advantageous against multiple neurological disorders, including Parkinson’s disease (PD) and cancer, because it can permeate the blood–brain barrier and regulate tumor cell growth, senescence, differentiation, apoptosis, and survival [11,12,13]. Sphingomyelin belongs to the group of phospholipids and sphingolipids found in high concentrations in the plasma membrane and the membranous myelin sheath surrounding the nerve cell axons. Naturally, these lipids are essential for impulse transmission, location of neurotransmitter receptors, myelin sheath, and blood–brain barrier (BBB) integrity. Their importance in cell signaling is mainly due to their ease of hydrogen bond formation with other molecules [14,15]. Sphingomyelin-based liposomes were reported to be an efficient carrier system due to their ability to cross the BBB and reduce accumulation. These liposomes are metabolized by the spleen and liver, permitting clearance. In addition to sphingomyelin, cholesterol (Chol) was added to enhance the circulatory time in vivo, with improved pharmacokinetics and therapeutic properties [16]. These lipids have been found to establish cholesterol/sphingomyelin-enriched nanodomains in the organelle membranes, which play a pivotal role in neurotransmitter release, synaptic plasticity, and regulating synaptic functions [17]. These properties benefit the development of innovative therapeutic systems with excellent loading capacities. Such loading and reducing options can be obtained in the biological synthesis of the SLNPs.

This study exploits *Ginkgo biloba* (*G. biloba*) leaf extracts (GBE). The BBB is composed of a class of G protein-coupled receptors (GPCRs), referred to as adenosine receptors (ARs), which essentially regulate the permeability of the BBB [18]. In response to this phenomenon, the GBE possess ginkgolides A, B, C, bilobalide, and kaempferol, which can effectively activate the A1R, rendering the BBB more permeable while inducing cellular changes such as a decrease in trans-endothelial electrical resistance, an increase in the formation of actinomyosin stress fibers and the alteration of the tight junctions. Furthermore, the introduction of GBE provides a reversible opening of the BBB for four hours, preventing dysregulation in the homeostasis of the brain [19].

The SLNPs alone may not adequately bind small interfering ribonucleic acid (siRNA). Hence, the highly cationic homopolypeptide poly-L-lysine (PLL) was used to add stability to the SLNP and to bind the anionic siRNA. PLL has been used in early studies to deliver nucleic acids but was challenged with short circulation times in vivo [20,21], limiting their use in gene delivery. The use of PLL to functionalize NPs for the delivery of deoxyribonucleic acid (DNA) [22] and messenger ribonucleic acid (mRNA) [23] has since been widely utilized. The delivery of therapeutic siRNA has been commonly investigated in gene silencing experiments. However, its efficiency depends on reaching its target site without being degraded in vivo. Naked siRNA is susceptible to nuclease digestion and can be removed by the mononuclear phagocytic system (MPS) [24]. Due to its size and anionic nature, cellular uptake can be problematic [25]. Hence, conjugating siRNA to delivery vehicles may protect them, enhance their circulation half-life, and improve cellular uptake and gene silencing. The current study used PLL-modified SLNPs to bind and condense the siRNA adequately.

This study aims to biologically synthesize the SLNPs utilizing GBE to create an efficient biocompatible gene delivery vehicle. This study further assesses the loading capabilities of the delivery vehicle for future siRNA-mediated gene therapy. The efficacy of the therapeutic nanosystem is evaluated in the human neuroblastoma (SH-SY5Y) and embryonic kidney (HEK293) cells to determine their applicability to neurodegenerative disorders such as PD. To date, a lack of information about the medical applicability of these NPs has been noted, necessitating further studies to highlight their vast capabilities.

## 2. Materials and Methods

### 2.1. Materials

Ethidium bromide, glycerol, bromophenol blue, xylene cyanol, ethylenediaminetetraacetic acid (EDTA) disodium salt, sodium dodecyl sulfate, phosphate-buffered saline (PBS) tablets, 3-(4,5-dimetylthiazol-2-yl)-2,5-diphenyltetrazolium bromide (MTT), dimethyl sulfoxide (DMSO) and RNase A were purchased from Merck (Darmstadt, Germany). Poly-L-lysine (PLL, 30–70 kDa), sphingomyelin, cholesterol, and dialysis tubing (MWCO 12 kDa) were obtained from Sigma-Aldrich (St. Louis, MO, USA). The Promega Corporation (Madison, WI, USA) supplied the luciferase assay system. Control, siGENOME non-targeting siRNA, and the MycoFluor™ Mycoplasma detection kit were provided by Thermo Scientific Dharmacon Products (Lafayette, CO, USA). Ultrapure agarose was purchased from Bio-Rad Laboratories (Hercules, CA, USA). Embryonic kidney (HEK293) and neuroblastoma (SH-SY5Y) cells were initially purchased from the American Type Culture Collection (ATCC, Manassas, VA, USA). Eagles Minimum Essential Medium (EMEM) with L-glutamine (4.5 g/L), Dulbecco’s modified Eagle medium/nutrient mixture F-12 (DMEM/F12), trypsin–versene mixture, and penicillin–streptomycin mixtures (10,000 U/mL) were purchased from Lonza BioWhittaker (Walkersville, MD, USA). Fetal bovine serum (FBS) was supplied by GIBCO, Life Technologies Ltd. (Inchinnan, UK). The Muse^®^ Caspase-3/7 kit was sourced from Luminex (Austin, TX, USA). Nest Biotechnologies (Wuxi, China) provided the sterile plasticware for cell culture. DNase/RNase-free water (Life Technologies, Carlsbad, CA, USA) and ultrapure water (18 Mohm) were used (Milli-Q50, Millipore, Guyancourt, France). All reagents were of analytical grade.

### 2.2. Sample Collection and Preparation

Young *G. biloba* leaves (Supplementary Figure S1) were collected within the vicinity of Durban, KwaZulu-Natal, South Africa, sealed in a plastic bag to inhibit transpiration and drying out during transport. After washing the leaves with distilled water, they were dried using a paper towel. Exactly 20 g of the leaves were carefully cut into pieces, added to a beaker containing distilled water (75 mL), and heated to 80–90 °C, stirring for 10 to 15 min. When the solution turned green/yellow, it was removed from the heat, filtered through a number 5 Whatman filter paper, and stored at −4 °C.

### 2.3. Synthesis of Solid Lipid Nanoparticles (SLNPs)

Sphingomyelin (10 mg/mL in methanol) and cholesterol (10 mg/mL in chloroform) were mixed at 50:50, 40:60, and 60:40 mol% and added to a round bottom flask (50 mL). The concentration of sphingomyelin was maintained at 2 mg/mL. The lipid mixture was rotary evaporated at 50 °C until a film was deposited on the bottom of the flask. This lipid film was vacuum-dried for 12 h to remove any residual solvents. The synthesized SLNPs were then divided into two equal aliquots. One aliquot was made up to 50 mL with deionized water (H_2_O-SLNPs), while 50 mL GBE (GBE-SLNPs) was added to the second aliquot. The water and the GBE were added dropwise with constant stirring at 60–70 °C. The mixtures were sonicated for 2 min and then centrifuged at 3000 rpm for 5 min to prevent aggregation.

### 2.4. Functionalization of SLNPs with Poly-L-Lysine (PLL)

A 1:1 ratio (PLL: SLNP) was used to produce PLL-SLNPs. The PLL was used as a stabilizing agent to impart a positive charge to the SLNPs for the subsequent siRNA binding.

### 2.5. Preparation of PLL-SLNP:siRNA Nanocomplexes

All PLL-SLNP formulations were briefly vortexed (1 min) and then sonicated (15 min) before use. To obtain different mass (*w/w*) or N/P (+/−) ratios, various volumes of the SLNP suspensions were combined with a fixed amount of siRNA (0.5 μg). The suspensions were adjusted to a final volume of 10 μL using HEPES-buffered saline (HBS) and incubated for 1 h at room temperature to facilitate nanocomplex formation. All nanocomplexes were freshly prepared before each experiment.

### 2.6. Characterization

The SLNPs and PLL-SLNPs were assessed over a 400–800 nm wavelength range using a Biomate 3 spectrophotometer (Thermo Scientific, CO, USA). The spectrum for the PLL was utilized to indicate the successful conjugation [26,27,28,29].

Fourier transform infrared (FTIR) spectroscopy was performed on a Perkin Elmer Spectrum 100 FTIR spectrophotometer (PerkinElmer Inc., Waltham, MA, USA) fitted with a universal attenuated total reflection (ATR) sampling accessory. All samples were freeze-dried, and 10 mg of each sample was analyzed from 4000 to 400 cm^−1^ with 64 co-added scans and a resolution of 4 cm^−1^. The IR spectra were plotted using the OriginLab (2023b) software (version 10.5.0 OriginLab Corporation, Northampton, MA, USA).

The ultrastructural morphology of the SLNPs and their siRNA-based nanocomplexes (at optimum binding ratios) were examined by transmission electron microscopy (TEM) using a Jeol T-1010 TEM (JEOL Ltd.; Tokyo, Japan). A Soft Imaging Systems MegaView III side-mounted 3-megapixel digital camera was used to capture the images of the SLNPs and their nanocomplexes.

The hydrodynamic size and the zeta potential of the SLNPs and nanocomplexes were obtained by dynamic light scattering (DLS) using the Malvern Zetasizer Nano-ZS (Malvern Instruments Ltd., Worcestershire, UK). Diluted samples (1:100, 1 mL in 18 MΩ H_2_O) at room temperature were analyzed.

### 2.7. Intercalation Assay

The intercalating agent ethidium bromide (EtBr) was used to investigate the ability of the PLL-SLNPs to bind and compact the siRNA [30]. EtBr (2 μL, 100 μg/mL) in 100 μL of HBS was added to a well in a 96-well flat-bottomed black FluorTrac plate (Greiner Bio-One, Frickenhausen, Germany). This provided a baseline fluorescence (0%) measured at excitation and emission wavelengths of 520 nm and 610 nm, respectively (GloMax^®^ -Multi Detection System, Promega BioSystems, Madison, WI, USA). After that, 1 μL of the target siRNA (0.3 μg/μL) was added, and the fluorescence was recorded as 100%. The PLL-SC-SLNPs were introduced in aliquots of 1 μL, and the fluorescence was recorded until an inflection point or plateau was reached. Equation (1) was used to calculate the relative fluorescence (FR).

(1) $$FR\left(\%\right)=\left(\frac{Fi-F0}{Fmax-F0}\right)\times 100$$

where Fi = fluorescence reading following the sequential addition of the SLNPs, F0 = reading of the EtBr, and Fmax = reading after siRNA addition.

### 2.8. Band-Shift Assay

The siRNA-binding capability of the PLL-SLNPs was assessed using a band-shift assay [30]. When the nanocomplex migration is fully retarded, an electroneutral complex is formed, indicating that the positive charges on the SLNPs have completely neutralized the negative charges of the siRNA. This complex fails to migrate through the gel matrix. Nanocomplexes were prepared as previously described. After complex formation, 2 μL of gel loading buffer (50% glycerol, 0.05% bromophenol blue, 0.05% xylene cyanol) was added, and the nanocomplexes were subjected to agarose gel electrophoresis using a 2% (*w*/*v*) agarose gel containing 1 μg/mL of EtBr. Electrophoresis was carried out at room temperature or 30 min at 50 V. The gels were visualized under UV300 transillumination, and the images were captured using a Vacutec Syngene G: Box BioImaging system (Syngene, Cambridge, UK).

### 2.9. Protection Assay

The protection afforded by the SLNPs to the bound siRNA against enzymatic degradation was investigated following RNase A-mediated digestion [30]. This assay was used to determine the siRNA’s compaction by the SLNPs and to observe if the siRNA will maintain its integrity during delivery in an in vivo system. As determined from the band-shift assay, nanocomplexes were prepared at the suboptimum, optimum, and supra-optimum binding mass ratios (*w*/*w*). Following incubation (1 h), RNase A was introduced to each sample to obtain a final concentration of 10% (*v*/*v*). Two controls were used in this study: a positive control (siRNA only) and a negative control (siRNA treated with RNase A). Samples were incubated for 2 h at 37 °C. EDTA (10 mM) and SDS (0.5%) were added after incubation, followed by a 20 min incubation at 55 °C. Agarose gel electrophoresis was then undertaken as completed for the band shift assay.

### 2.10. Cell Culture

Both cell lines were used in their second or third passages after purchase and monitored routinely using an inverted microscope. Cells were grown at 37 °C in 5% CO_2_ into 25 cm^2^ flasks and subcultured into multiwell plates for the cell culture assays. Routine mycoplasma detection was conducted before experimentation using the MycoFluor™ Mycoplasma Detection Kit. Cells were all mycoplasma clear before cell-based assays were performed.

### 2.11. Cytotoxicity Studies

HEK293 and SH-SY5Y cells were subcultured into clear 96-well plates at a density of 1.8 × 10^5^ cells per well and incubated overnight at 37 °C. Nanocomplexes (in triplicate) were prepared using SLNPs at the previously chosen ratios using 50 nM siRNA (0.067 μg). A positive control consisting of untreated cells was used to represent 100% cell survival. Following incubation, the old medium was replenished with fresh medium (EMEM supplemented with 10% FBS and 1% antibiotics). The nanocomplexes were then introduced to the cells and incubated over 48 h at 37 °C. Thereafter, the medium was replaced with 10% MTT solution (5 mg/mL in PBS) in 100 μL of EMEM. The cells were incubated for an additional 4 h at 37 °C. The MTT-containing EMEM was removed, and 100 μL of DMSO was pipetted into each well. Absorbance was determined at 570 nm using a Mindray MR-96A microplate reader (Vacutec, Hamburg, Germany), with DMSO as the blank. The cell viability was quantified using the following equation:

$$Cell\;Viability\left(\%\right)=\left(\frac{Absorbance\;of\;Treated\;Cells}{Absorbance\;of\;Control}\right)\times 100$$

### 2.12. Caspase 3/7 Activity

The Muse™ Caspase-3/7 kit was utilized to quantify the apoptotic status and the permeability of the cell membrane at various stages of apoptosis, using caspases-3/7 activity and a dead cell dye [29]. The cells (1.8 × 10^5^ cells per well) were prepared as completed for the MTT assay and treated with the respective nanocomplexes. Following a 48 h incubation, the cells were rinsed with PBS, trypsinized, and 1x assay buffer (50 µL) was introduced. The mixture was mixed by vortexing, followed by the addition of caspase 3/7 (5 µL). The cells were incubated at 37 °C for 30 min, after which caspase 7-AAD working solution (150 µL) was added. The cells were incubated away from light at room temperature for 5 min. Caspase activity was assessed using a Muse™ cell analyzer (Luminex, TX, USA).

### 2.13. Cellular Uptake Studies

The cellular uptake assay was performed to examine the potential of the PLL-SLNPs to traverse the cellular membrane and localize in the nucleus. The assay used the BLOCK-iT™ fluorescent oligo to provide a means of visualization through its ability to fluoresce. Cells (1.8 × 10^5^ cells per well) were prepared and treated as completed for the cytotoxicity assay, except for using 1 µL BLOCK-iT™ fluorescent oligo (1 mM and 2 mM) instead of the siRNA, which was incubated for 1 h with the SLNPs. The treated cells were incubated overnight. The media was discarded, and PBS (2 × 60 µL) was used to wash the cells. Cells were viewed under an Olympus CKX41 inverted phase contrast fluorescence microscope (Olympus Corporation, Tokyo, Japan) at an excitation and emission wavelength of 494 nm and 519 nm, respectively. The cells were thereafter lysed with 80 µL 1x lysis buffer with gentle rocking at 30 rpm for 15 min on a STR 6 platform shaker (Stuart Scientific, Staffordshire, UK). Following this, the cells were dislodged from the wells, and the cell lysates were placed into a 96-well black plate, and the fluorescence was quantified using a Glomax multi-detection system (Promega Biosystems, CA, USA). Protein concentrations were evaluated using the standard bicinchoninic (BCA) assay. The fluorescence readings were normalized against the BCA assay results and expressed as relative fluorescent units (RFU)/mg protein.

### 2.14. Statistical Analysis

Data are reported as means ± standard deviation (SD, *n* = 3). Statistical analysis of the mean values was conducted using one-way ANOVA, followed by Dunnett multiple comparison post hoc test. Statistical analyses were carried out with a 95% confidence interval (CI), and results were considered significant if the *p*-value was less than 0.05 (*p* < 0.05). Statistical data were collected using GraphPad Prism 9 (GraphPad Inc. San Diego, CA, USA).

## 3. Results

### 3.1. UV–Vis and FTIR Spectroscopy

**Figure 1:**
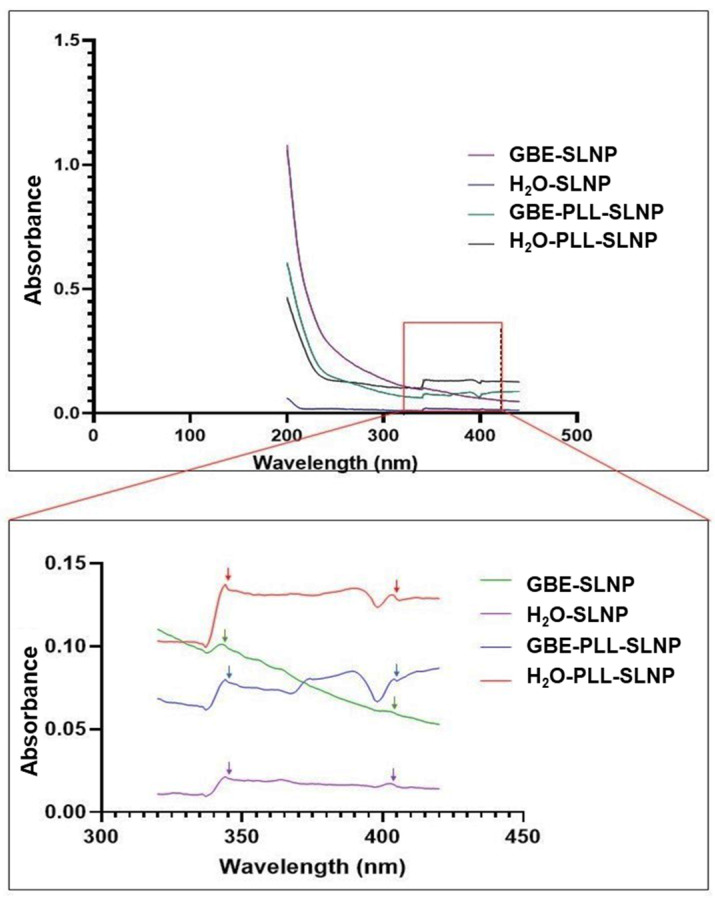
UV–vis spectra of GBE-SLNPs, H_2_O-SLNPs, GBE-PLL-SLNPs, and H_2_O-PLL-SLNPs. The amplified 320–420 nm section shows the peak and trough variations that are indicated by the arrows.

While there is a lack of prior literature on the specific absorbance characteristics of SLNPs, the UV–vis spectroscopy data presented here confirm the successful conjugation with PLL and highlight distinct differences between GBE-based and H_2_O-based SLNPs (Figure 1). The absorbance maxima listed in Table 1 reveal a hypochromic (blue) shift for the H_2_O-based SLNPs and a bathochromic (red) shift for the GBE-based SLNPs, consistent with the expected effects of PLL conjugation on the NP surface. The second peak for both conjugated and unconjugated SLNPs displayed a hypochromic shift, further supporting the occurrence of surface modifications attributable to PLL. Additionally, a single trough appeared at 400 nm and 397 nm for the PLL-conjugated SLNPs, reinforcing the idea that the observed spectral shifts indicate successful conjugation rather than instrumental artifacts.

**Figure 2:**
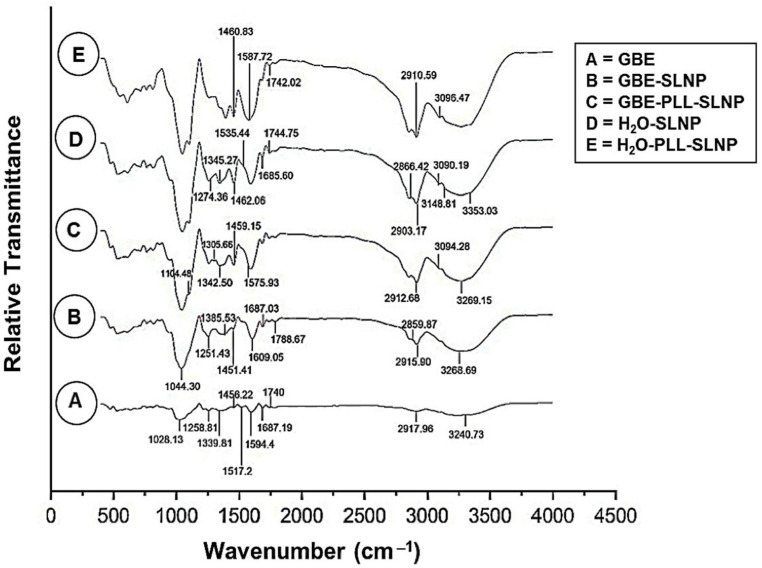
FTIR spectra of: (**A**) GBE, (**B**) GBE-SLNPs, (**C**) GBE-PLL-SLNPs, (**D**) H_2_O-SLNPs, and (**E**) H_2_O-PLL-SLNPs.

FTIR analysis confirmed the successful bio-synthesis of the SLNPs and the conjugation of poly-L-lysine (PLL) (Figure 2, Table 2). The broad peak observed at 3297.30 cm^−1^ in the GBE sample, corresponding to O-H stretching, is retained in the GBE-SLNPs and GBE-PLL-SLNPs with slight shifts, indicating successful encapsulation of GBE within the SLNP matrix [31]. Characteristic peaks for C-H stretching vibrations observed at 2918.90 cm^−1^ in GBE are present in both GBE-SLNPs and GBE-PLL-SLNPs, confirming the incorporation of GBE’s aliphatic components [32]. The presence of PLL is confirmed by the N-H stretching vibrations (3094.28 cm^−1^) in GBE-PLL-SLNPs, characteristic of the amide groups in PLL [29,33,34]. The C=O stretching vibrations at 1742.15 cm^−1^ in GBE-PLL-SLNPs and H_2_O^−^ PLL-SLNPs indicate strong hydrogen bonding and electrostatic interactions between PLL and the lipid components of the SLNPs [32,35].

### 3.2. TEM and DLS

**Figure 3:**
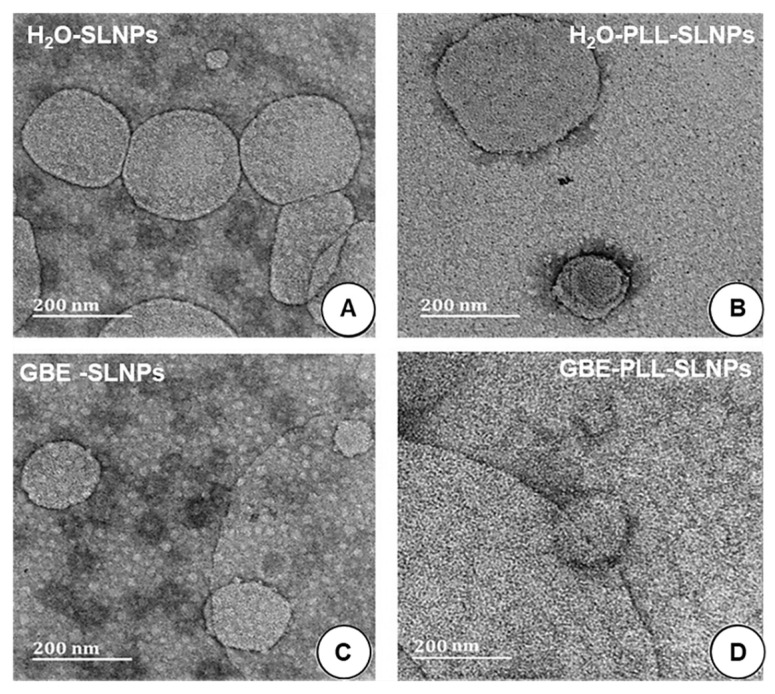
TEM micrographs of the SLNPs. (**A**) H_2_O-SLNPs, (**B**) H_2_O-PLL-SLNPs, (**C**) GBE-SLNPs, (**D**) GBE-PLL-SLNPs. Scale bar = 200 nm.

All SLNPs were spherical, and most were below 200 nm in size (Figure 3), except for H_2_O-PLL-SLNPs (Figure 3B), where some SLNPs were above 200 nm under TEM. The GBE-synthesized SLNPs were smaller, with sizes below 120 nm. Upon SLNP conjugation with PLL, no apparent morphological changes were noted. There are observable differences in size in Figure 3B, which can be attributed to the binding of PLL to the SLNPs. However, despite these variations, the overall average size of the NPs was observed to be 140 nm.

The SLNPs and corresponding nanocomplexes were subjected to DLS to assess their hydrodynamic sizes and zeta potentials to determine their colloidal stability. The results (Table 3) portrayed a size range of less than 200 nm, suitable for biomedical applications. The sizes obtained from the TEM were noted to be lower than the hydrodynamic sizes obtained from DLS.

The GBE-SLNPs showed better stability than the H_2_O-based SLNPs, which could be due to the natural properties of the GBE in reducing and stabilizing the SLNPs. The zeta potentials increased (>35 mV) upon conjugation to PLL. The PLL-SLNPS exhibited good stability upon binding to siRNA, promoting their role as suitable nanocarriers. The polydispersity index indicated uniform and monodispersed SLNPs with a lack of aggregation (PDI < 0.1) [40,41].

### 3.3. Intercalation Assay

**Figure 4:**
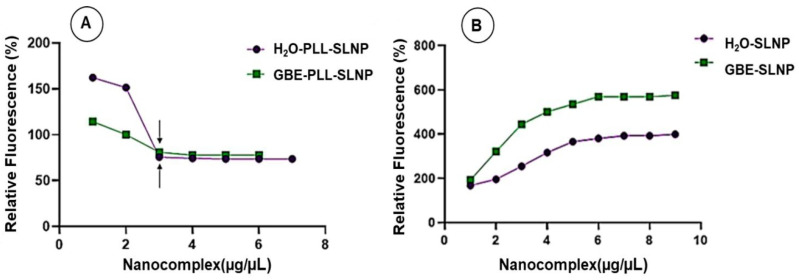
Ethidium bromide intercalation assay of (**A**) GBE- and H_2_O-PLL-SLNPs and (**B**) GBE- and H_2_O-SLNPs with siRNA (0.3 μg/μL). Arrows indicate the inflection points in (**A**).

This assay assessed the PLL-SLNPs’ ability to effectively compact the siRNA, preventing premature dissociation from the nanocarrier. This assay is based on the intercalation of EtBr within the siRNA, which fluoresces. The sequential addition of the SLNPs disturbs this intercalation, causing fluorescence quenching until a plateau is attained (Figure 4A). The GBE-PLL-SLNPs portrayed a slightly better compaction ability (77.78%) than their H_2_O-based counterparts (73.60%). As expected, the non-functionalized SLNPs (Figure 4B) (SLNPs without the PLL) could not bind the siRNA. This led to a steady increase in the relative fluorescence, with no fluorescence quenching noted.

### 3.4. Band Shift Electrophoresis and Protection Assay

**Figure 5:**
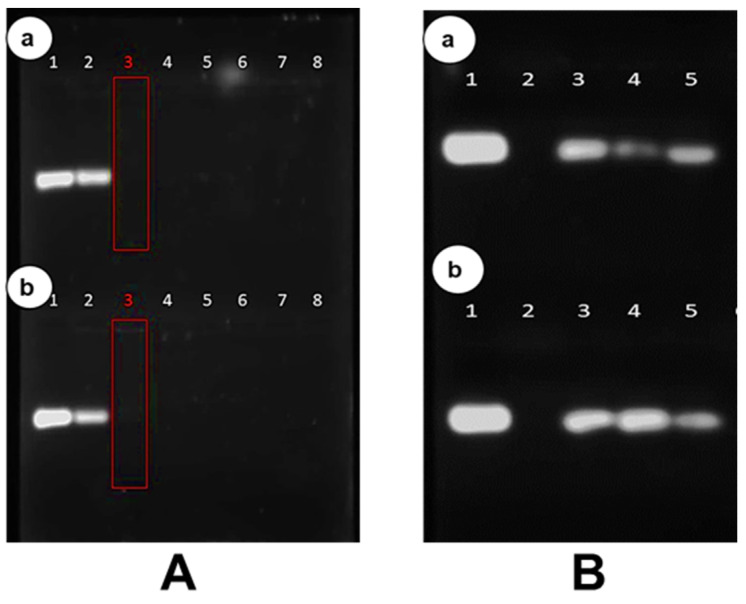
(**A**) The band shift assay. (**a**) GBE-PLL-SLNPs: Lanes 1–8 (0, 0.2, 0.4, 0.6, 0.8, 0.10, 0.12, 0.14 µg) and (**b**) H_2_O-PLL-SLNPs: Lanes 1–8 (0, 0.1, 0.2, 0.3, 0.4, 0.5, 0.6, 0.7 µg). The siRNA was maintained at 0.5 µg. The red boxes indicate the optimum binding ratios, superseded by the supra-optimum ratio and preceded by the sub-optimum ratio. (**B**) The RNase protection assay. In both (**a**,**b**), Lanes 1 and 2 contain the positive (undigested siRNA) and negative (digested siRNA) controls. (**a**) GBE-PLL-SLNPs: Lane 3–5 (0.2, 0.4, 0.6 µg) and (**b**) H_2_O-PLL-SLNPs: Lanes 3–5 (0.1, 0.2, 0.3 µg). All nanocomplexes were complexed with the targeted siRNA (0.5 µg).

The band shift assay determines the complete binding of the siRNA by the cationic SLNPs due to electrostatic interactions. The optimum binding ratio is established when no free siRNA migrates into the gel, which occurs at a point of electroneutrality [30]. The red box depicts the optimum ratio (*w*/*w*) of the PLL-SLNPs (Figure 5A). The PLL-SLNPs suspended in distilled water bound the siRNA at a slightly lower ratio of 0.2:1 (*w*/*w*) compared to the GBE-PLL-SLNPs with a ratio of 0.4:1 (*w*/*w*). Both PLL-SLNPs showed good binding abilities with no distinct advantage of either method. The differences in binding efficiencies can be attributed to the surface charge of the PLL-SLNPs, as a greater positive charge would lead to a better binding potential. This will affect the final binding ratios.

This assay was repeated after 12 months (Supplementary Figure S2) to establish the integrity of the SLNPs after storage at 4 °C. The same binding ratios were acquired, suggesting that these SLNPs have a good shelf-life and are relatively stable with no degradation during storage.

The protection of the siRNA by the SLNPs is crucial for safe and efficient delivery to the target cells. This assay provides a means of simulating the host environment to determine the extent of protection. The band intensities varied across the ratios. However, the SLNPs protected the siRNA from RNase A digestion compared to the negative control (naked siRNA in lane 2), which was degraded and not visible on the gel (Figure 5B). As previously, this assay was also re-examined after 12 months (Supplementary Figure S3), with similar protection abilities noted, suggesting the good stability of these nanocomplexes.

### 3.5. Cytotoxicity Studies

**Figure 6:**
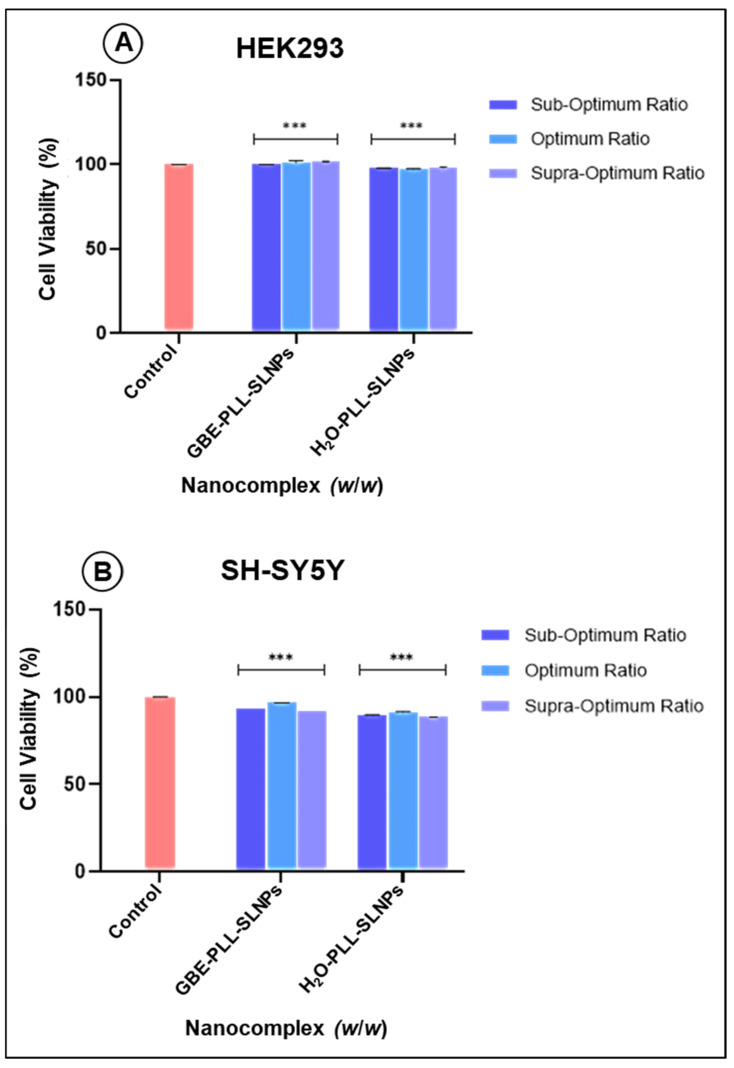
The MTT cytotoxicity assay in (**A**) HEK293 and (**B**) SH-SY5Y cells. Data are presented as means ± standard deviation (*n* = 3). A significant difference was observed between the treated cells and the control group (*** *p* < 0.001; Tukey’s multiple comparisons test).

The MTT colorimetric assay determines cell viability based on the mitochondrial dehydrogenase activity [42]. The HEK293 cells (cell viability > 90%) were able to tolerate all the nanocomplexes, with the supra-optimum ratio of the GBE-PLL-SLNPs portraying enhanced cellular viability (101.76%) (Figure 6A). There was no statistically significant difference in the cytotoxicity between the GBE- and H_2_O-based SLNPs. Similarly, the neuroblastoma SH-SY5Y cells maintained high viability, above 85% (*p* < 0.001) (Figure 6B).

### 3.6. Caspase 3/7 Activity

**Figure 7:**
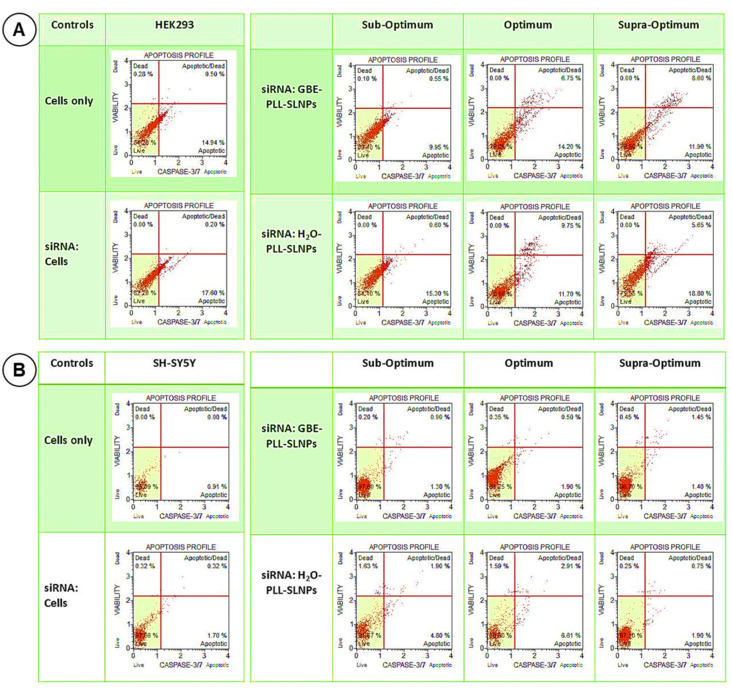
Caspase 3/7 activity induced by the nanocomplexes at the three studied ratios in the (**A**) HEK293 and (**B**) SH-SHY5Y cells. The cytographs depict the apoptotic responses of the (**B**) cells following treatment with the PLL-SLNPs at the sub-optimum, optimum, and supra-optimum ratios.

The results obtained from the cytotoxicity assay were correlated to the caspase 3/7 assay. The cell survival in the HEK293 cells was high (>75%) with minimal apoptosis (Figure 7A), similar to the MTT assay. In the SH-SY5Y cells (Figure 7B), <11.1% of the cells displayed any apoptotic behavior. Overall, the results correlated closely to that of the MTT assay, with high cell survival noted in the HEK293 and SH-SY5Y cells, suggesting the safety and suitability of these NPs as therapeutic nanocarriers. The apoptosis rate was assessed further (Supplementary Figure S4), with no significant differences observed between the treatment groups. This suggested that both GBE-PLL-SLNPs and H_2_O-PLL-SLNPs did not induce significant apoptosis in these cells.

### 3.7. Cellular Uptake

The BLOCK-iT™ fluorescent oligo was used to surrogate the therapeutic siRNA and demonstrated significant differences in cellular uptake efficiencies between the tested formulations. Nanocomplexes were formulated at 1:1 and 2:1 (*w*/*w*) concentrations of oligo to the SLNPs to ensure binding optimization. The fluorescent images for the HEK293 cells are shown in Supplementary Figure S5.

**Figure 8:**
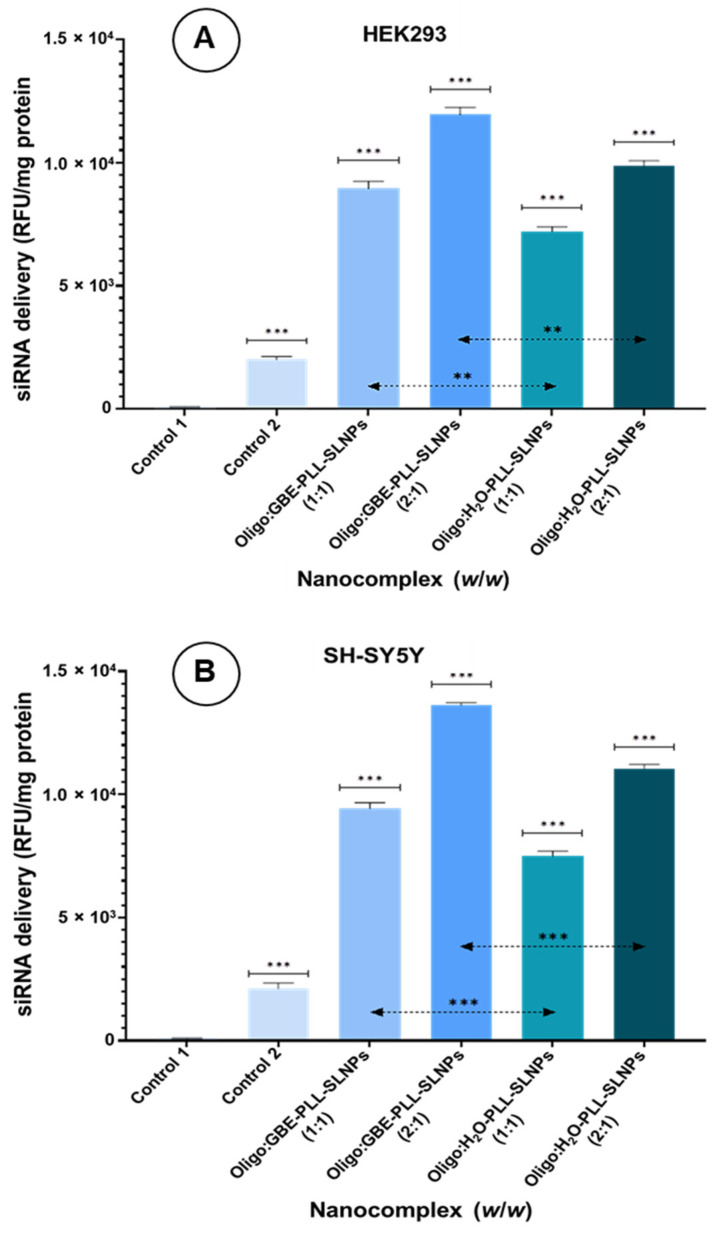
(**A**) HEK293 cells and (**B**) SH-SY5Y cells treated with BLOCK-iT™ fluorescent oligo (0.5 and 1.0 µg) conjugated to PLL-SLNPs at 1:1 and 2:1 (*w*/*w*) ratios. Intracellular fluorescence was measured after 24 h. (**A**) Control 1 = HEK293 cells only, and control 2 = naked/uncomplexed BLOCK-iT™ fluorescent oligo. Controls were compared to the nanocomplexes (*** *p* < 0.001) and further compared with the two treatment groups as indicated by the dotted line (** *p* < 0.005). (**B**) Control 1 = SH-SY5Y cells only, and control 2 = naked BLOCK-iT™ fluorescent oligo. Controls were compared to the nanocomplexes and further compared with the two treatment groups as indicated by the dotted line (*** *p* < 0.001). Statistically significant differences in the therapeutics were noted in both (**A**,**B**).

In the HEK293 cells, the naked oligo control exhibited low fluorescence (>2000; *p* < 0.001) (Figure 8A). Treatment with GBE-PLL-SLNPs at the 1:1 ratio resulted in moderate-high intensity fluorescence (>8000; *p* < 0.001), while the 1:2 ratio showed significantly greater fluorescence (>11,000). The SLNPs were visualized primarily in the cytoplasm and around the nuclear region. The H_2_O-PLL-SLNPs displayed a similar trend in fluorescence intensity from the 1:1 to 1:2 ratio but with reduced cellular uptake (Figure 8A). Notably, SLNP aggregation near the nucleus was observed in cells treated with the 1:2 ratio.

The fluorescent images for the SH-SY5Y cells are shown in Supplementary Figure S6. A similar trend was seen in the SH-SY5Y cells (Figure 8B), with greater uptake at the 1:2 ratios for both SLNPs. The GBE-PLL-SLNPs showed better fluorescence at the 1:2 ratio (13,623.52; *p* < 0.001) than at the 1:1 ratio (9431.11; *p* < 0.001). The H_2_O-PLL-SLNPs exhibited lower overall cellular uptake. Furthermore, the GBE-PLL-SLNPs demonstrated a more uniform distribution and internalization, whereas the H_2_O-based nanocomplexes showed less uniformity.

The addition of GBE in SLNP formulation was validated due to its superior cellular uptake and distribution, possibly facilitated by the bioactive compounds in GBE, such as flavonoids and terpenoids, which enhanced the interaction between SLNPs and cellular membranes. This will enable a higher intracellular concentration of the siRNA cargo and improve the overall therapeutic efficacy.

## 4. Discussion

UV–vis and FTIR spectroscopy confirmed the successful formulation and functionalization of the SLNPs. The observed spectral shifts are consistent with molecular changes caused by PLL conjugation and the presence of GBE rather than detector noise or artifacts. The low signal intensity in the 300–400 nm range, though close to the noise threshold, aligns with previous findings that lipid-based nanoparticles generally exhibit low intrinsic absorbance due to limited chromophoric content and particle size distribution [34,43]. These shifts suggest that PLL conjugation modifies the electronic environment on the NP surface, producing hypochromic or bathochromic shifts due to changes in surface interactions [44,45].

The stability of the GBE-PLL-SLNP complex highlights its potential as a promising drug or gene delivery vehicle. This stability is primarily supported by specific chemical interactions and the presence of active centers within the NP matrix, which facilitate binding and maintain the structural integrity of the complex under physiological conditions. The GBE-based SLNPs exhibited a bathochromic shift, possibly due to the bioactive compounds such as flavonoids and terpenoids that are present in GBE, which can affect surface charge and polarizability. Other GBE components, such as ginkgolides, can interact with the NP’s surface through stabilizing interactions, namely, hydrogen bonding and π–π stacking, resulting in changes to optical properties [46]. Prior studies have shown that such components stabilize NPs and contribute to distinct spectral shifts [7,47], supporting the interpretation that the observed shifts in our study reflect true surface interactions due to PLL and GBE functionalization.

Additionally, the combination of PLL and GBE introduces multiple active centers in the SLNPs, which strengthen the NP structure and enhance its therapeutic-carrying capabilities. The PLL, due to the protonated amine groups on its lysine residues, imparts a positive charge, facilitating electrostatic interactions with the negatively charged siRNA. This cationic structure acts as an active center, effectively condensing the siRNA onto the NP surface and forming a stable nanocomplex. This is crucial for protecting the siRNA from enzymatic degradation in systemic circulation [7,45]. Such stable conjugations are essential, as they reduce premature dissociation and degradation of the siRNA before it can reach the target cell.

The flavonoids and terpenoids not only contribute to NP stability but also improve biocompatibility and the NP’s carrier potential. Additionally, the antioxidant properties of GBE reduce oxidative stress around the nanoparticles, adding a protective layer in cellular environments [43]. The antioxidative effects also help prevent aggregation and degradation, supporting the SLNPs’ morphology and functionality over time [47].

Besides size, ultrastructural morphology and polydispersity are essential for cellular uptake. TEM determined the morphology and size of the SLNPs in a dry state, while DLS assessed them in an aqueous state, which attributed to the difference in sizes obtained using these two methods [40,41]. This could also be due to the SLNPs coming together as clusters in the aqueous environment, which were recorded as larger particles under DLS [43]. Such aggregation is possible due to electrostatic interactions between the PLL with anions in solution or hydrophobic interactions between the lipid components [48,49]. The PLL-GBE-SLNPs appeared larger under TEM compared to the size obtained from DLS. This discrepancy is a common observation when comparing the dry-state TEM preparation and aqueous-based DLS measurements. TEM provides individual particle dimensions, often revealing slight flattening or aggregation on the grid, while DLS measures the hydrodynamic diameter, including the surrounding solvation layer in the solution. Such differences are well-documented studies and highlight the complementary relationship between TEM and DLS [40,41,44].

Furthermore, the average size is based on the scattered light intensity, which is inherently more sensitive to larger particles due to the r6 dependence of the scattering intensity on the radius. Consequently, a low number of aggregates could disproportionately affect the average size [49]. While there was a noticeable increase in size, the SLNPs retained their integrity and properties. The appended PLL condensed the SLNPs to a greater extent, which was also reported previously for gold and selenium NPs [22,50]. GBE has been reported to improve the stability of gold [50], copper [51], silver [47], and palladium [19] NPs. The PDI values were all low and well below 0.3. This confirmed the high levels of monodispersity of the SLNPs and their nanocomplexes [40,41], which was further evidenced by a lack of aggregation in the TEM images. The SLNPs exhibited favorable sizes and zeta potentials for cellular uptake.

Upon PLL conjugation, a higher positive charge was noted for the SLNPs, which was beneficial for siRNA binding and interaction with the anionic cellular membrane [27,29]. Good compaction of the siRNA was noted, which is essential to prevent its premature dissociation from the SLNP. Successful binding of the siRNA to the SLNPs can be attributed to the cationic structure of PLL, allowing its protonated terminal lysine residues and the anionic phosphate groups of the siRNA to interact electrostatically [29,52], forming compact nanocomplexes. A recent study reported a similar result using PLL-functionalized gold NPs compared to the unfunctionalized and polyethylene glycol-containing NPs [29]. This compaction efficacy is vital to their in vitro and in vivo protective ability. This higher compaction for the GBE-based SLNPs may be further due to the functional groups (hydroxyl, carbonyl) from the flavonoid and terpenoids of the extract that interact with the siRNA through hydrogen bonding and electrostatic interactions.

Lipid-mediated siRNA delivery is often challenged by unfavorable interactions with serum nucleases, leading to their degradation before they reach their target site. The SLNPs protected the siRNA from RNase A digestion, suggesting their stability in vivo. The SDS used in this study released most of the siRNA from the nanocomplexes, which did not lose their integrity. However, as reported previously, SDS can sometimes show incomplete nucleic acid release [22,30,52]. The MTT cytotoxicity and caspase 3/7 assays confirmed the low cytotoxicity of the SLNPs in the HEK293 and SH-SY5Y cells. Caspases 3/7 are known to execute apoptosis directly and contain a cleavable peptide substrate, Asp-Gly-Val-Asp (DEVD), which helps monitor apoptosis [29]. The MTT and caspase 3/7 assays showed similar trends in both cell lines. The low cytotoxicity can be attributed to the antioxidant properties of GBE, which reduce oxidative stress. The terpenoids and flavonoids in GBE scavenge free radicals and upregulate antioxidant enzymes, providing cytoprotective effects [53]. This counteracts the oxidative stress induced by free siRNA. GBE has been shown to modulate signaling pathways such as the PI3K/Akt pathway, which is actively involved in cell survival, contributing to its protective effects. Modulating the MAPK/ERK pathway protects neuronal cells from apoptosis [46,54]. GBE also reduces the expression of caspases and Bax (pro-apoptotic proteins) while enhancing the expression of anti-apoptotic proteins (e.g., Bcl-2) [46,53].

This is crucial for PD studies, where oxidative stress and apoptosis are critical factors in the degeneration of dopaminergic neurons. The good cell viability and reduced apoptotic profiles in the GBE-PLL-SLNP formulations could provide neuroprotective effects in PD models, suggesting promise as a therapeutic agent. This is achieved through the anti-inflammatory effects of GBE, which inhibit the activation of microglia and astrocytes, key players in neuroinflammation. Wang et al. (2021) demonstrated a 15% reduction in cytotoxicity in lung A549 cells with lower lactate dehydrogenase (LDH) release [55], while Liu et al. (2020) reported a significant decrease in apoptosis and ROS levels in PC12 cells using GBE-incorporated NPs [56]. These studies corroborate the present study’s results and highlight the GBE-formulated SLNPs’ protective abilities.

The Caspase 3/7 assay provided further insights into cellular viability and apoptosis. While the MTT assay measures metabolic activity and is commonly used as an indicator of cell viability, it does not exclusively measure cell survival. Early apoptotic cells retain metabolic activity and continue to reduce MTT, leading to possible overestimation of viability [57]. Elevated caspase 3/7 activity is a hallmark of apoptosis and provides an indicator of cells undergoing programmed cell death, regardless of their metabolic status [58]. Thus, combining the MTT and caspase 3/7 assays allowed us to more accurately distinguish between live, metabolically active cells and cells in the early stages of apoptosis.

In terms of the neuroprotective effects attributed to GBE, previous studies support its role in mitigating oxidative stress and reducing apoptotic pathways, particularly in neuronal cell models [46]. GBE can scavenge free radicals and inhibit caspase activation, which could provide protective effects against neurotoxicity by stabilizing mitochondrial membranes and reducing ROS production [59]. Although our primary objective was to assess cell viability and apoptosis, these properties of GBE suggest that our nanocomplexes may confer neuroprotective effects, as indicated by the low apoptotic activity in the caspase 3/7 assay.

Cellular uptake was assessed using the BLOCK-iT™ fluorescent oligo, a fluorescein-labeled, double-stranded RNA duplex, mimicking a siRNA molecule’s standard length, configuration, and charge. This molecule is modified to increase stability, permitting the measurement of a fluorescent signal over an extended period. This oligo localizes mainly to the nucleus [60] and can be assessed by measuring the fluorescence. It has been proposed that cholesterol within nanocomplexes could interact with lipids and specific receptors in the cell membrane [61], or it may be internalized by membrane fusion [62]. Hence, the siRNA can be delivered into the cytoplasm, escaping the endolysosomes. This is important since these intracellular vesicles often lead to the loss of siRNA, with less than 2% of siRNA entering the cytoplasm. This occurs immediately after cellular uptake and before endosomal maturation and endolysosomal fusion, which results in the degradation of the entrapped siRNA [63]. The cellular uptake studies showed favorable uptake of the SLNPs into the HEK293 and the neuroblastoma SH-SY5Y cells used as the Parkinsonian model. The GBE-PLL-SLNPs were more efficiently taken up by the cells than the H_2_O-PLL-SLNPs and also proposed a dose-dependent nature of internalization. The bioactive compounds enhanced the interaction between the SLNPs and the cellular membrane by integrating into the lipid bilayers, increasing membrane fluidity and permeability, and facilitating the uptake of the SLNPs [64]. GBE stimulates endocytosis due to ginkgolides, which modulate the activity of endocytic pathways, leading to increased internalization of the GBE-PLL-SLNPs. This process allows the siRNA to reach the cytoplasm and exert its therapeutic effects [65].

Flavonoids (quercetin, kaempferol, isorhamnetin) also interact with cell surface receptors, which include the epidermal growth factor (EGFR) and the vascular endothelial growth factor receptors (VEGFR), triggering signaling pathways that enhance endocytosis and internalization of NPs [65]. Terpenoids (ginkgolides, bilobalide) in GBE can modulate receptors involved in signaling, promoting receptor-mediated endocytosis [66]. Recent studies showed that GBE could enhance cellular uptake by modulating oxidative stress and inflammation pathways [67,68].

These studies further support our findings that GBE-based SLNPs offer good cellular uptake and therapeutic benefits. Overall, our results describe a safe, biocompatible, and efficient nano-delivery system capable of delivering therapeutics to Parkinsonian cells while maintaining exceptional cellular viability. However, a few limitations were identified in this study. First, the experiments were conducted in vitro using the HEK293 and SH-SY5Y cell lines, which may not fully replicate the complexity of an in vivo environment. Hence, further in vivo studies are necessary to evaluate the efficacy and safety of these GBE-PLL-SLNPs in a more complex biological system, including interactions with the blood–brain barrier. There is a need to examine if any immune responses were produced in response to the nanocomplexes. While the study demonstrated efficient siRNA binding and delivery, the long-term effects and stability of these SLNPs within the cellular environment were not assessed. Analyzing intracellular siRNA release and localization can provide insights into the delivery mechanism and the subsequent gene-silencing efficacy. These limitations highlight the need for further studies to fully establish the therapeutic potential of GBE-PLL-SLNPs for PD.

## 5. Conclusions

The present study described the successful synthesis, characterization, cytotoxicity, and cellular uptake of the PLL-SLNPs with and without GBE. The synergism between the components of the SLNPs provided a highly efficient vehicle for the safe delivery of the siRNA. The SLNPs were well tolerated in the embryonic kidney and the neuroblastoma cells, as evidenced by the cytotoxicity and the caspase 3/7 assays. The use of the GBE further enhanced their protective ability, reducing apoptosis while improving cell viability. Although this study had positive outcomes, more evidence to support the benefits of using the GBE is needed. This study provides proof of concept for using PLL-SLNPs in gene delivery, warranting further investigation into their therapeutic potential in examining gene silencing in the Parkinson’s cell model.

## Figures and Tables

**Table 1:** Peaks and troughs exhibited by the SLNPs.

Nanoparticles	Peak 1	Peak 2	Trough
**SLNPS**	343 nm	401 nm	-
**PLL-SLNPS**	345 nm	402 nm	400 nm
**GBE-SLNPS**	345 nm	401 nm	-
**GBE-PLL-SLNPS**	341 nm	404 nm	397 nm

**Table 2:** The wavenumbers (cm^−1^) associated with the respective functional groups as confirmed by FTIR [31,32,33,35,36,37,38,39].

Functional Group	Wavenumber (cm^−1^)	Interpretation
**O-H**	3297.30	Hydroxyl groups in phenolic compounds
**C-H**	2918.90	Methyl and methylene groups of lipids
**C=O**	1789.10	Carbonyl groups
**C=C**	1674.00	Alkene groups
**N-H**	1595.40	Amide groups in proteins or peptides
**C-O-C**	1258.00	Ester and ether linkages
**O-H**	3268.69	Incorporation of phenolic compounds from GBE
**C-H**	2915.90, 2859.87	Aliphatic chains in sphingomyelin and cholesterol
**C=O**	1788.67	Hydrogen bonding, successful encapsulation
**C-O**	1251.53	Presence of esters and ethers from GBE
**N-H**	3094.28	Conjugation of PLL to SLNPs
**C-H**	2912.68	Aliphatic chains in lipids and PLL
**C=O**	1742.15	Conjugation of PLL to GBE
**C-N**	1305.66	Amine groups from PLL
**O-H**	3353.03	Hydration of lipid components
**N-H**	3148.81, 3090.19	Presence of sphingomyelin within SLNPs
**C-H**	2903.17	Aliphatic chains in cholesterol and sphingomyelin
**C=O**	1744.75	Ester groups in the SLNP matrix
**N-H**	3096.43	Interaction between PLL and the lipid matrix
**C=O**	1742.01	Electrostatic interactions between lipids and PLL

**Table 3:** TEM and DLS nanoparticle and nanocomplex sizes, zeta potential, and polydispersity indices (PDI).

NPs	TEM	DLS					
		Nanoparticle			Nanocomplex		
	Size (nm ± SD)	Size (nm ± SD)	Zeta Potential (mV) Mean ± SD (*n* = 3)	PDI	Size (nm ± SD)	Zeta Potential (mV) Mean ± SD (*n* = 3)	PDI
H_2_O-SLNPs	180.2 ± 25.3	190.5 ± 13.9	13.1 ± 1.1	0.003	-	-	-
H_2_O-PLL-SLNPs	140.0 ± 1.0	120.4 ± 7.1	35.6 ± 0.3	0.013	139.5 ± 35.2	43.3 ± 0.1	0.037
GBE-SLNPs	118.3 ± 15.6	122.4 ± 12.7	24.4 ± 1.3	0.012	-	-	-
GBE-PLL-SLNPs	136.3 ± 1.0	115.3 ± 10.7	36.8 ± 1.3	0.010	128.3 ± 20.3	45.4 ± 0.8	0.029

## Data Availability

The authors declare that all data from this study are reported in the manuscript and supplementary material. Further information is available from the corresponding author upon request.

## Supplementary Materials

The following supporting information can be downloaded at: https://www.mdpi.com/article/10.3390/polym16233265/s1, Supplementary Figure S1. *Ginkgo biloba* leaves used in the extraction process (Photograph by author K. Jagaran). Figure S2: The band shift assay after 12 months of storage of the SLNPs at 4 °C. (A) GBE-PLL-SLNPs: Lanes 1–8 (0.2, 0.4, 0.6, 0.8, 0.10, 0.12, 0.14 µg) and (B) H_2_O-PLL-SLNPs: Lanes 1–8 (0, 0.1, 0.2, 0.3, 0.4, 0.5, 0.6, 0.7 µg). The siRNA was kept constant at 0.5 µg. The red and yellow arrows indicate the optimum binding ratios, superseded by the supra-optimum ratio and preceded by the sub-optimum ratio. Figure S3: Agarose gel images of the RNase protection assay after 12 months of storage of the SLNPs at 4 °C. In both (A) and (B), Lanes 1 and 2 contain positive and negative controls. (A) GBE-PLL-SLNPs: Lane 3–5 (0.2, 0.4, 0.6 µg) and (B) H_2_O-PLL-SLNPs: Lanes 3–5 (0.1, 0.2, 0.3 µg). All nanocomplexes were complexed with targeted siRNA (0.5 µg). Supplementary Figure S4. Apoptosis rates in HEK293 and SH-SY5Y cells following treatment with the siRNA: PLL-SLNP nanocomplexes at the sub-optimum, optimum, and supra-optimum (*w*/*w*) ratios. Apoptosis levels were determined from the recorded caspase 3/7 activity. No statistical significance (ns) was observed among all groups, indicating that each treatment maintained the initial apoptosis rate, supporting the safety of the treatments. Supplementary Figure S5. Fluorescent images showing cellular uptake in the HEK293 cells. (A) Control of HEK293 cells not treated with the fluorescent oligo, (B) Control of naked Block-IT™ oligo not complexed to the SLNPs, (C) Oligo:GBE-PLL-SLNPs in a 1:1 (*w*/*w*) ratio; (D) Oligo:GBE-PLL- SLNPs in a 2:1 (*w*/*w*) ratio; (E) Oligo:H_2_O-PLL-SLNPs in a 1:1 (*w*/*w*) ratio; (F) Oli-go:H_2_O-PLL-SLNPs in a 2:1 (*w*/*w*) ratio. The cells were visualized at 100x magnification. Scale Bar = 200 µm. Supplementary Figure S6. Fluorescent images showing cellular uptake in the SH-SY5Y cells. (A) Control of SH-SY5Y cells not treated with the fluorescent oligo, (B) Control of naked Block-IT™ oligo not complexed to the SLNPs, (C) Oligo:GBE-PLL-SLNPs in a 1:1 (*w*/*w*) ratio; (D) Oligo:GBE-PLL- SLNPs in a 2:1 (*w*/*w*) ratio; (E) Oligo:H_2_O-PLL-SLNPs in a 1:1 (*w*/*w*) ratio; (F) Oligo:H_2_O- PLL-SLNPs in a 2:1 (*w*/*w*) ratio. The cells were visualized at 100x magnification. Scale Bar = 200 µm.
