# Supplementary material for: Bio-Inspired Polymeric Solid Lipid Nanoparticles for siRNA Delivery: Cytotoxicity and Cellular Uptake In Vitro
Source: Polymers (Basel). 2024 Nov 24;16(23):3265. doi: 10.3390/polym16233265 (PMC11644305; doi:10.3390/polym16233265)
Supplement: Supplementary file 1 [file polymers-16-03265-s001.zip › polymers-3293488-supplementary.pdf]

polymers

MDPI

Supplementary Material

Bio-Inspired Polymeric Solid Lipid Nanoparticles for siRNA Delivery: Cytotoxicity and Cellular Uptake In Vitro

Keelan Jagaran, Saffiya Habib and Moganavelli Singh *

Nano-Gene and Drug Delivery Laboratory, Discipline of Biochemistry, University of KwaZulu-Natal, Private Bag X54001, Durban, 4000, South Africa; 215055447@stu.ukzn.ac.za (KJ), saffiya.habib@gmail.com (SH)
* Correspondence: singhm1@ukzn.ac.za, Tel.: +2731-2607170.

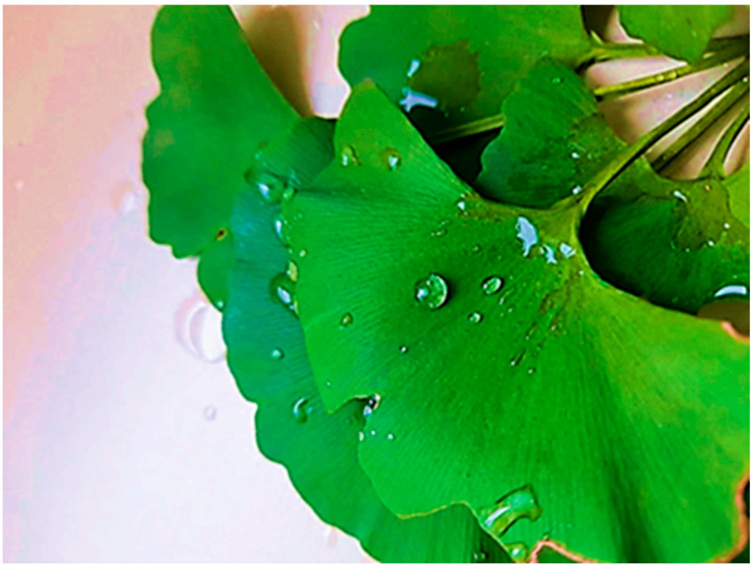

Supplementary Figure S1. *Ginkgo biloba* leaves used in the extraction process (Photograph by author K. Jagaran).

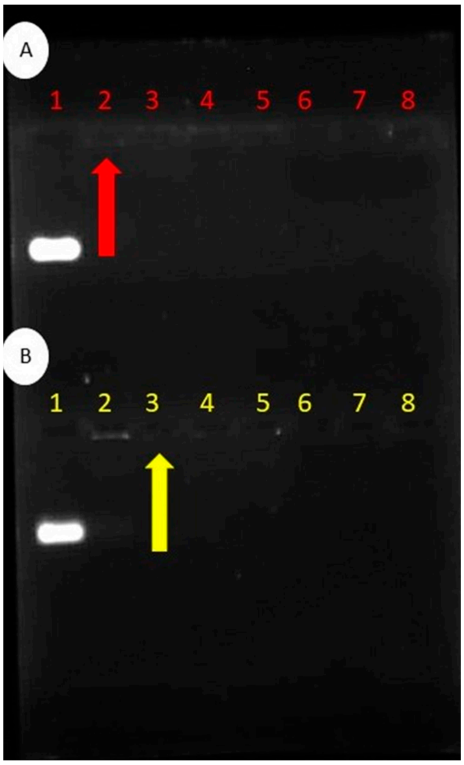

Supplementary Figure S2. The band shift assay after 12 months of storage of the SLNPs at 4 °C. (A) GBE-PLL-SLNPs: Lanes 1 – 8 (0.2, 0.4, 0.6, 0.8, 0.10, 0.12, 0.14 µg) and (B) H_2O-PLL- SLNPs: Lanes 1-8 (0, 0.1, 0.2, 0.3, 0.4, 0.5, 0.6, 0.7 µg). The siRNA was kept constant at 0.5 µg. The red and yellow arrows indicate the optimum binding ratios, superseded by the supra-optimum ratio and preceded by the sub-optimum ratio.

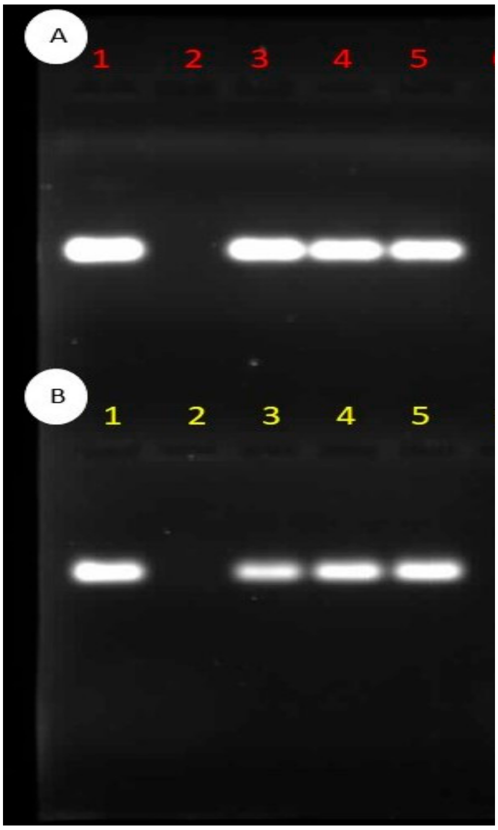

Supplementary Figure S3. Agarose gel images of the RNase protection assay after 12 months of storage of the SLNPs at 4 °C. In both (A) and (B), Lanes 1 and 2 contain positive and negative controls. (A) GBE-PLL-SLNPs: Lanes 3-5 (0.2, 0.4, 0.6 µg) and (B) H_2O-PLL-SLNPs: Lanes 3 – 5 (0.1, 0.2, 0.3 µg). All nanocomplexes were complexed with targeted siRNA (0.5 µg).

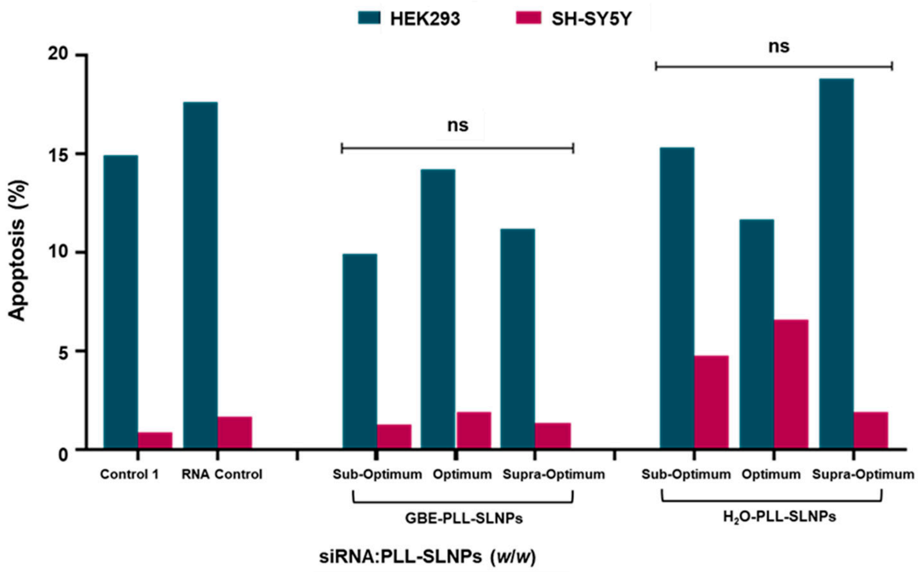

Supplementary Figure S4. Apoptosis rates in HEK293 and SH-SY5Y cells following treatment with the siRNA: PLL-SLNP nanocomplexes at the sub-optimum, optimum, and supra-optimum (w/w) ratios. Apoptosis levels were determined from the recorded caspase 3/7 activity. No statistical significance (ns) was observed among all groups, indicating that each treatment maintained the initial apoptosis rate, supporting the safety of the treatments.

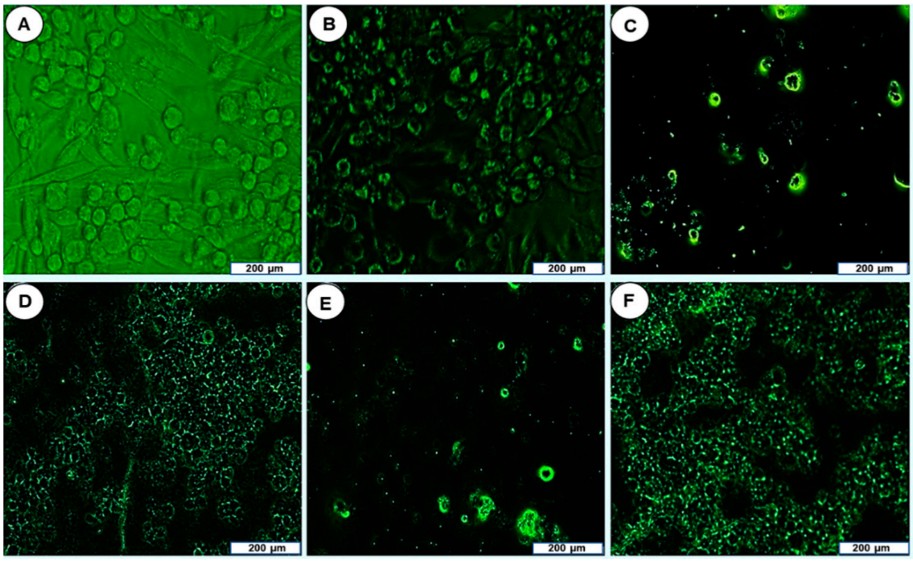

Supplementary Figure S5. Fluorescent images showing cellular uptake in the HEK293 cells. (A) Control of HEK293 cells not treated with the fluorescent oligo, (B) control of naked Block-IT™ oligo not complexed to the SLNPs, (C) Oligo:GBE-PLL-SLNPs in a 1:1 (w/w) ratio; (D) Oligo:GBE-PLL- SLNPs in a 2:1 (w/w) ratio; (E) Oligo:H_2O-PLL-SLNPs in a 1:1 (w/w) ratio; (F) Oligo:H_2O-PLL-SLNPs in a 2:1 (w/w) ratio. The cells were visualized at 100x magnification. Scale Bar = 200 µm.

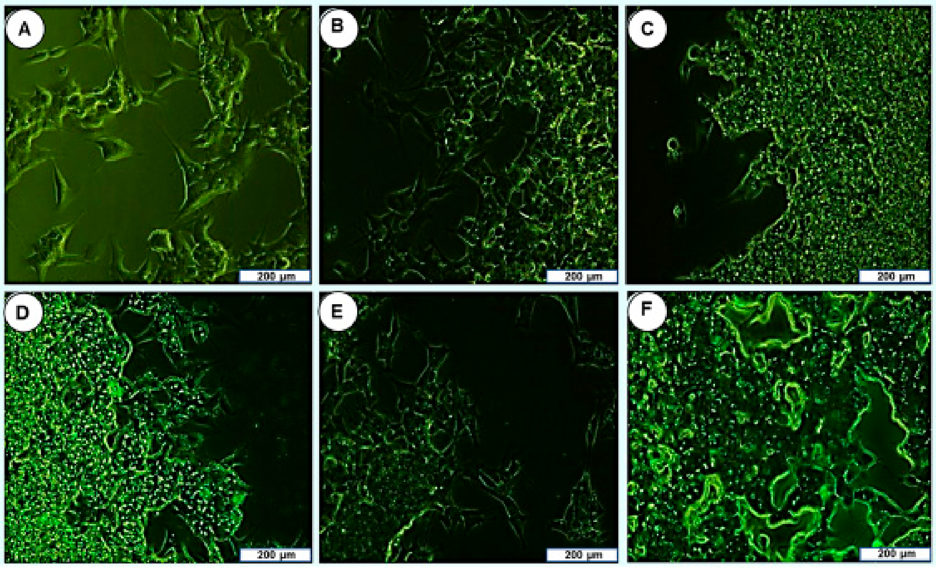

Supplementary Figure S6. Fluorescent images showing cellular uptake in the SH-SY5Y cells. (A) Control of SH-SY5Y cells not treated with the fluorescent oligo, (B) control of naked Block-IT™ oligo not complexed to the SLNPs, (C) Oligo:GBE-PLL-SLNPs in a 1:1 (w/w) ratio; (D) Oligo:GBE-PLL- SLNPs in a 2:1 (w/w) ratio; (E) Oligo:H_2O-PLL-SLNPs in a 1:1 (w/w) ratio; (F) Oligo:H_2O- PLL-SLNPs in a 2:1 (w/w) ratio. The cells were visualized at 100x magnification. Scale Bar = 200 µm.

Disclaimer/Publisher's Note: The statements, opinions and data contained in all publications are solely those of the individual author(s) and contributor(s) and not of MDPI and/or the editor(s). MDPI and/or the editor(s) disclaim responsibility for any injury to people or property resulting from any ideas, methods, instructions or products referred to in the content.
